# Green tea polyphenols and Tai Chi for bone health: Designing a placebo-controlled randomized trial

**DOI:** 10.1186/1471-2474-10-110

**Published:** 2009-09-04

**Authors:** Chwan-Li Shen, Ming-Chien Chyu, James K Yeh, Carol K Felton, Ke T Xu, Barbara C Pence, Jia-Sheng Wang

**Affiliations:** 1 Department of Pathology, Texas Tech University Health Sciences Center, Lubbock, Texas, USA; 2 Laura W Bush Institute for Women's Health, Texas Tech University Health Sciences Center, Lubbock, Texas, USA; 3 Department of Laboratory Science and Primary Care, Texas Tech University Health Sciences Center, Lubbock, Texas, USA; 4 Department of Cell Physiology and Molecular Biophysics, Texas Tech University Health Sciences Center, Lubbock, Texas, USA; 5 Department of Nutrition, Texas Tech University, Lubbock, Texas, USA; 6 Department of Mechanical Engineering, Texas Tech University, Lubbock, Texas, USA; 7 Department of Health, Exercise, and Sport Sciences, Texas Tech University, Lubbock, Texas, USA; 8 Graduate Healthcare Engineering Option, Texas Tech University, Lubbock, Texas, USA; 9 Applied Bench Core Laboratory, Winthrop-University Hospital, Mineola, New York, USA; 10 Department of Obstetrics and Gynecology, Texas Tech University Health Sciences Center, Lubbock, Texas, USA; 11 Department Family and Community Medicine, Texas Tech University Health Sciences Center, Lubbock, Texas, USA; 12 Department of Environmental Health Science, University of Georgia, Athens, Georgia, USA

## Abstract

**Background:**

Osteoporosis is a major health problem in postmenopausal women. Evidence suggests the importance of oxidative stress in bone metabolism and bone loss. Tea consumption may be beneficial to osteoporosis due to its antioxidant capability. However, lack of objective data characterizing tea consumption has hindered the precise evaluation of the association between tea ingestion and bone mineral density in previous questionnaire-based epidemiological studies. On the other hand, although published studies suggest that Tai Chi (TC) exercise can benefit bone health and may reduce oxidative stress, all studies were conducted using a relatively healthy older population, instead of a high-risk one such as osteopenic postmenopausal women. Therefore, this study was designed to test an intervention including green tea polyphenol (GTP) and TC exercise for feasibility, and to quantitatively assess their individual and interactive effects on postmenopausal women with osteopenia.

**Methods/Design:**

One hundred and forty postmenopausal women with osteopenia (defined as bone mineral density T-score at the spine and/or hip between 1 to 2.5 SD below the reference database) were randomly assigned to 4 treatment arms: (1) placebo group receiving 500 mg medicinal starch daily, (2) GTP group receiving 500 mg of GTP per day, (3) placebo+TC group receiving both placebo treatment and TC training (60-minute group exercise, 3 times per week), and (4) GTP+TC group receiving both GTP and TC training for 24 weeks. The outcome measures were bone formation biomarker (serum bone alkaline phosphatase), bone resorption biomarker (serum tartrate resistant acid phosphatase), and oxidative DNA damage biomarker (urinary 8-hydroxy-2'-deoxyguanosine). All outcome measures were determined at baseline, 4, 12, and 24 weeks. Urinary and serum GTP concentrations were also determined at baseline, 4, 12, and 24 weeks for bioavailability. Liver function was monitored monthly for safety. A model of repeated measurements with random effect error terms was applied. Traditional procedures such as ANCOVA, chi-squared analysis, and regression were used for comparisons.

**Discussion:**

We present the rationale, design, and methodology of a placebo-controlled randomized trial to investigate a new complementary and alternative medicine strategy featuring a dietary supplement and a mind-body exercise for alleviating bone loss in osteopenic postmenopausal women.

**Trial registration:**

ClinicalTrials.gov identifier: NCT00625391

## Background

Osteoporosis is a degenerative bone disease characterized by low bone mass and structural deterioration of bone tissue, leading to bone fragility and an increased susceptibility to fractures, especially of the hip, spine, and wrist [1]. Women are four times more likely to develop osteoporosis than men because of a decrease in estrogen level after menopause in conjunction with generally lighter and thinner bones [2]. The rapid decrease in bone mineral density (BMD) that occurs in the first 3 to 5 years immediately following menopause, and the slower decrease that continues throughout the remainder of a women's life, markedly increase the risk of a hip or vertebral fracture, which is a major cause of morbidity and mortality in older women [1,2].

Osteoporosis is associated with many etiological causes, such as poor nutrition, cytokines, hormones, and aging. Reactive oxygen species (ROS) are considered to be responsible for the aging process [3] and contributing to etiology of various degenerative diseases, including osteoporosis [4-6]. It is believed that the rate of bone formation gradually diminishes, while the rate of bone resorption is unaltered or accelerated with advancing age in humans, resulting in net bone loss leading to the development of osteoporosis. Estrogen is a phenolic compound which enables it to detoxify accumulated ROS [7]. Thus, estrogen deficiency, which occurs after menopause, leads to bone loss through increased osteoclastic function, and represents the major pathological determinant responsible for postmenopausal bone loss, as ROS stimulate osteoclasts that are responsible for bone resorption [7,8].

There is evidence that ROS are involved in bone resorption with a direct contribution of osteoclast-generated superoxide to bone degradation [9,10], and oxidative stress increases differentiation and function of osteoclasts [11]. In addition, osteoblasts produce antioxidants such as glutathione peroxidase to protect against ROS [12], and they also produce transforming growth factor-β, which is involved in bone resorption [13]. Recent studies showed that oxidative stress inhibited osteoblastic differentiation [14] via extracellular signal-regulated kinases (ERK) and ERK-dependent nuclear factor-κB signaling pathways [6].

A possible relationship between tea drinking and osteoporosis was suggested by a number of studies [15]. The bioactive components in tea may benefit bone health in terms of maintaining higher BMD [16-21] and reducing fracture risk [22,23]. The health benefits of tea consumption in preventing cancers and cardiovascular diseases have been intensively investigated [24]. However, limited information is available about the protective effect of consumption of tea or its bioactive components on bone health. These published results on BMD and tea consumption were all based on cross-sectional studies or retrospective studies and are, therefore, inconsistent [25], which may compromise the quality of evidence.

The most widely recognized properties of tea polyphenols, extract forms of tea, are their antioxidant activities, arising from their ability to scavenge ROS [26]. Tea polyphenols also bind to metal ions, preventing these ions from participating in peroxidative reactions. Green and black tea and isolated tea polyphenols have been shown to scavenge reactive oxygen and nitrogen species, and to reduce their damage to lipid membranes, proteins and nucleic acids in cell-free systems [26]. Among different green tea components, green tea polyphenols (GTP) have been shown to be safe and to have many beneficial health effects in various human populations [27]. Several mechanisms have been proposed for the osteo-protective effect of GTP including decreasing oxidative stress [28,29], increasing activity of antioxidant enzymes [28], and decreasing expression of proinflammatory mediators [28,29] in rodent models.

However, no study has ever been conducted to show the antioxidant role of GTP in bone metabolism of postmenopausal women with low bone mass.

Exercise has been shown to benefit osteoporosis by reducing bone loss. Numerous studies have reported the effect of exercise on BMD, particularly in postmenopausal women with or without osteopenia/osteoporosis [30-32]. Tai Chi (TC) exercise has gained much attention recently in the rehabilitation and geriatric community [32-35]. The systematic study of TC's effect on bone mass, however, is limited. A case-control study conducted in postmenopausal Chinese women in Hong Kong showed that women having done TC for more than 4 years had significantly higher BMD in the lumbar spine, proximal femur, and ultradistal tibia than non-exercising women [32]. Their 12-month (without intervention) follow-up measurement showed generalized bone loss in both groups, but the TC exercisers had a significantly slower decrease in BMD than the non-exercising group. A longitudinal study showed that 12 months of 108-form TC decelerated bone loss in early postmenopausal women compared to sedentary controls [35]. A cross-sectional study revealed that regular TC exercise may be associated with higher BMD [32] in early postmenopausal women than in sedentary controls [34]. Among these previous studies, there was only one longitudinal study [35], but that study did not target a high-risk population (osteopenia or osteoporosis). Besides, that study employed a complicated 108-form TC, which was difficult for the U.S. population to learn. The two previous cross-sectional studies [32,35] could not identify the type of TC (among dozens currently being practiced worldwide) that had been practiced by the women included in the study.

Exercise can have positive or negative effects on oxidative stress depending on training load, training specificity and the basal level of training [36]. High-intensity exercise increases oxidative stress, whereas moderate exercise tended to decrease oxidative stress [37]. TC has been characterized as an exercise of moderate intensity [38,39]. TC couples muscular activity with an internally directed focus, producing a temporary self-contemplative mental state. Such internal focus is in contrast to conventional body-centered aerobic and muscular fitness exercise, in which there is little or no mindful component [40]. Moreover, both TC and Yoga are considered as mind-body exercise [41], and both of these have been used as mind-body therapies in the treatment of musculoskeletal disorders with implications for the elderly [41]. Yoga has been demonstrated to reduce oxidative stress by showing a significantly lower level of free radicals and a higher level of superoxide dismutase in blood compared to the sedentary control [42]. However, no study has demonstrated the effect of TC on oxidative stress.

Therefore, the objective of the present study was to test complementary and alternative medicine interventions including GTP and TC exercise for feasibility, and to quantitatively assess their individual and interactive effects on osteopenic postmenopausal women. We hypothesized that 24 weeks of GTP supplement, TC exercise, and their combination would benefit bone remodeling, as measured by bone biomarkers in postmenopausal women with osteopenia, compared to those receiving placebo only, and the changes in bone biomarkers associated with bone remodeling would be correlated with the changes in oxidative stress. This hypothesis was based on the premise that both TC and GTP may reduce oxidative stress, and that oxidative stress has been demonstrated to adversely affect bone health by both increasing differentiation and function of osteoclasts [11] and inhibiting osteoblastic differentiation [14].

In this paper, we present the design and detailed protocol of a placebo-controlled and randomized trial, as well as a discussion of the overall challenges of conducting this trial. The results from this trial will be reported at the completion of the study in accordance with the Consolidation of Standards for Reporting Trials guidelines [43].

## Methods/Design

### Study design

The combination of dietary supplement and moderate intensity exercise now becomes a new strategy in alleviating bone loss in postmenopausal women with low bone mass, due to the possible synergistic effects of the combination.

**Figure 1:**
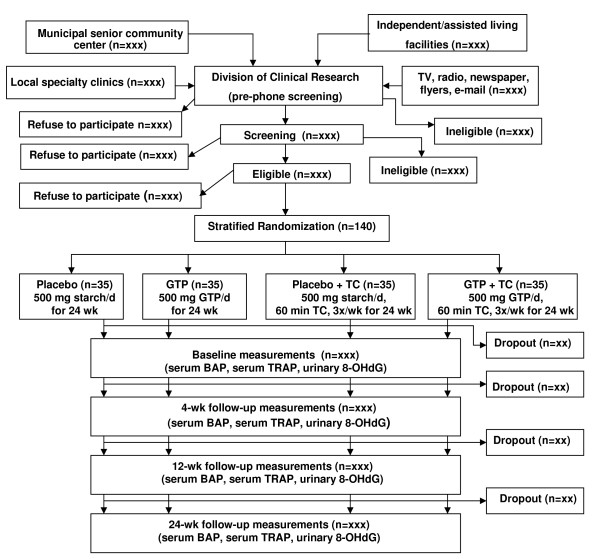
**Study flow chart**.

This was a 24-week placebo-controlled and randomized intervention trial to investigate the effects of GTP and TC on relevant primary and secondary endpoints in postmenopausal women with osteopenia. Women at least 2 years after menopause, with osteopenia, were recruited primarily from local senior independent/assisted living facilities, municipal senior community centers, and obstetrics and gynecology clinics. An initial sample size of 140 participants with an expected attrition rate of 15% over 24 weeks of intervention produced a final sample size of 120 participants. This sample size yielded a power of approximately 0.85 to 0.9 at α = 0.05 for detecting differences in primary and secondary outcome measures. After screening, qualified participants were matched for age and were randomly assigned to one of the four treatment groups: placebo, GTP, placebo+TC, and GTP+TC. During the 24-week intervention, all participants were provided with 500 mg elemental calcium and 200 IU vitamin Ddaily. The participants in the placebo group received 250-mg capsules of medicinal starch, 2 per day for 24 weeks. The GTP participants received 250-mg capsules of GTP, 2 per day for 24 weeks. The placebo+TC participants received both placebo and TC intervention (60-minute TC group class, 3 times per week) for 24 weeks. The GTP+TC participants received both GTP and TC intervention for 24 weeks. Participants received the primary and secondary outcome measures at baseline, 4, 12, and 24 weeks. The outcome measures were concentrations of bone biomarkers (serum bone-specific alkaline phosphatase, BAP and serum tartrate-resistant acid phosphatase, TRAP) and a biomarker of oxidative stress DNA damage (urinary 8-OHdG concentration). Urinary and serum GTP concentrations were determined at baseline, 4, 12, and 24 weeks. Liver function was also monitored monthly by assessing the activity of aspartate aminotransferase (AST) and alanine aminotransferase (ALT). Investigators evaluating the endpoints were blinded to intervention allocation. (See Study Flow Chart in Figure 1). The study has received Ethics approval from the Texas Tech University Health Sciences Center Institutional Review Board.

### Study population and recruitment

The present study was conducted in Lubbock, Texas and the surrounding area. This study consisted of women at least 2 years after menopause, ambulatory, irrespective of ethnicity/race, primarily recruited from local clinics, municipal senior community centers, and health fairs. Advertisement through local radio, TV, newspapers, senior newsletters/family magazines, flyers, and posters also proved to be very effective as an initial recruiting strategy.

### Screening

Assessment of potential participants involved a brief survey on menstrual history, medical history, availability for the study period, most recent BMD result if available, any existing bone metabolic diseases, etc., conducted over the phone by nurse research coordinators. This pre-screening procedure identified the potential participants who were interested in the research and were qualified for further screening. After telephone pre-screening, potential subjects were invited to attend an informed consent session. After signing consents and Health Insurance Portability and Accountability Act (HIPAA) forms, participants completed a detailed questionnaire that was developed to collect demographic, health, and dietary information. Then, the potential participants were invited for bone density screening and fasting blood screening visits.

#### Inclusion criteria

1. Women at least 2 years after menopause with osteopenia [44]. Osteopenia was defined as bone mineral density T-score at the spine and/or hip between 1 to 2.5 SD below the young normal xex-mateched areal BMD of the reference database [44].

2. Normal function of thyroid, liver, and kidney.

3. Serum 25-hydroxy vitamin D ≥ 20 ng/mL.

#### Exclusion criteria

1. History of metabolic bone diseases or treatments that might influence bone turnover.

2. History of cancer except for superficial basal or squamous cell carcinoma of the skin that was treated, and other malignancies treated at least 5 years ago without any evidence of recurrence.

3. Uncontrolled intercurrent illness or physical condition that would be a contraindication to exercise.

4. Cognitive impairment as defined as Mini-Mental State Examination score of 24 or less [45].

5. Depression as defined as Yesavage self-rated Geriatric Depression Scale score at least 11 [46].

6. Unwilling to accept randomization.

### Sample size

Data from previous studies by our team [47,48] were used for the calculation of the minimum sample size. The outcome measures were serum BAP [47], serum TRAP, and urinary 8-OHdG [48]. The baseline measurements for control (placebo) and intervention groups (GTP, placebo+TC, GTP+TC) were assumed to be the same as the values in the previous studies. Intervention groups were expected to exhibit changes in outcome measures in the follow-up by a certain percentage (10% increase, 10% decrease, and 20% decrease for BAP, TRAP, and 8-OHdG, respectively), while the placebo group was expected to exhibit no changes throughout the study. In the power analysis, it was further assumed that the correlation between baseline and the follow-up measurements was 0.85, and the correlation between the follow-up measurements was 0.95. A balanced sample, viz. same sample size for the control and intervention groups, was obtained. The main comparisons were expected to be made as follows: placebo vs. GTP; placebo vs. placebo+TC; and placebo vs. GTP+TC. Therefore, a sample size of 30 for each group was needed. Taking into consideration an expected attrition rate of 15%, N = 35 were recruited for each group, or a total of 140 participants to start the study.

### Randomization

All participants who had passed the screening criteria participated in randomization where participants were assigned to one of the 4 treatment arms with a fixed randomized scheme. In order to minimize the consequent bias, a stratified randomization method was employed based on age (>65 or <65 years old), history of green tea consumption, and history of mind-body exercise to assign participants to placebo, GTP, placebo+TC, or GTP+TC group.

### Intervention

#### Purchasing and masking of study agents

Placebo and GTP of the same lot, respectively, were purchased from Zhejiang Yuxin Pharmaceutical Co., Ltd., China (IND no. 77,470 by FDA). Each placebo capsule of 250 mg medicinal starch contained no GTP ingredient at detectable levels. Each GTP capsule contained 250 mg GTP ingredient [46.5% of epigallocatechin-3-gallate (EGCG), 21.25% of epigallocatechin (ECG), 10% of epicatechin (EC), 7.5% of epicatechin-3-gallate (EGC), 9.5% of gallocatechin gallate (GCG), and 4.5% of catechin] and the purity was higher than 98.5%. The use of placebo for masking the control group was desirable in this study to keep respondents blinded to the GTP treatment assignment. Placebo capsules were made of the same size and color as the GTP capsule. The placebo group provided a comparison of blood and urinary outcome measures and served as a basis to assess GTP's effect.

#### Provision of calcium plus vitamin D supplement

Calcium (500 mg elemental Ca, as Oyster Shell)/vitamin D (200 IU as cholecalcifero) supplement daily was provided to all participants (GlaxoSmithKline, PA). Adequate Ca/vitamin D intake is essential for realizing the beneficial effects of dietary supplements and exercise on BMD [49,50]. According to reported data, the calcium intake for our target population was about 700 to 800 mg daily [51,52]. Our goal was to supplement our subjects with additional elemental calcium (500 mg) in order to ensure that they receive total recommended calcium of 1000 mg-1500 mg. Similarly, the vitamin D supplementation we provided was to complement what the subjects were assumed to have received from diet and sun exposure, in order to reach the total daily recommendation. Therefore, if we had provided Ca/vitamin D at a higher dose, the potential effect of GTP may have been masked, due to Ca/vitamin D's strong anti-resorptive effect.

#### Treatment arms

The present study had four intervention groups, placebo, GTP, placebo+TC, and GTP+TC. Provision of placebo to the control and TC only groups increased the accuracy of the interventions, since it kept respondents blinded to the GTP treatment assignment. Participants in the placebo treatment group were provided 500 mg of medicinal starch daily for 24 weeks. Participants were instructed to take 1 capsule containing 250 mg in the morning and another 1 capsule in the evening, after meals. Similar to those in the placebo treatment group, participants in the GTP treatment group were provided 500 mg (250 mg capsule × 2 per day) of GTP per day for 24 weeks. Participants in placebo+ TC group received placebo treatment and TC training for 24 weeks. In addition to 500 mg of medicinal startch daily, the TC training was held at the exercise hall of a local community center, for 60 minutes three times a week (on three nonconsecutive days), for 24 weeks. Each training session consisted of 10 minutes of warm-up exercise, 40 minutes of TC, and 10 minutes of cool-down exercise. The warm-up exercise featured movements of all major body joints that were involved in the subsequent TC exercise. Participants were taught by a qualified instructor the 24-move simplified Yang-style TC ^[120] ^for 40 minutes during each session. The cool-down exercise involved whole body relaxation and deep breathing. Make-up sessions were offered to participants if necessary. Participants in the GTP+TC group received both GTP treatment and TC training for 24 weeks.

### Dietary Intake, Physical Activity, and Concomitant Medication Assessment

A complete and accurate dietary assessment was needed to determine whether drastic dietary changes occurred during the intervention period that could affect outcome measures. A 3-day dietary intake log was collected at the baseline, mid, and final visits and analyzed by Nutritionist Pro (First Data Bank, Inc., CA) to determine usual dietary intake (including specific macro- and micro-nutrients) over time and deviations from baseline. A physical activity log was collected at the baseline, mid, and final visits to record any deviation from the usual activities. Concomitant medications/therapies (including over the counter and dietary supplements) were documented, including drug name and the reason for the medication/therapies throughout the study period.

### Blinding/unblinding

All investigators, measurement and site personnel were blinded throughout this study. However, it was impossible to keep the TC instructor and the pharmacist, who distributed placebo or GTP capsules, blinded. Investigators evaluating the endpoints were blinded to intervention allocation.

### Sample collection

Fasting blood and urine samples (morning after first empty of the bladder) were collected for both primary and secondary outcome measures. Blood was drawn from a superficial arm vein with a syringe, transferred to a vacutainer, allowed to clot at room temperature, centrifuged at 1500× g for 10 min within 2 hr of collection, and aliquoted from each collection. During the visit for blood sample collection, urine samples were also collected in acid-washed polyethylene containers. Urine was aliquoted from each collection. All aliquoted blood and urine samples were stored in -80°C freezers prior to biochemical analyses.

### Evaluation of Adherence

Adherence to the intervention was evaluated by monitoring the consumption of capsules by pill count and attendance at TC training sessions. Compliance was determined as the percentage of all capsules ingested or possible group classes attended. The TC class attendance record was kept for each participant in the placebo+TC and GTP+TC groups.

### Evaluation of Adverse Events

Adverse effects associated with placebo or GTP treatment were self-reported by the participants if any, and by monitoring liver function monthly in the course of the intervention trial. Participants in the placebo+TC and the GTP+TC groups were also queried about any adverse events during TC training sessions by self-reported phone calls. All observed or volunteered adverse events, regardless of suspected causal relationship to the study treatments, were recorded on the adverse event form throughout the study.

### Measurements

Every participant was evaluated at baseline (prior to starting intervention), 4, 12, and 24 weeks of intervention.

#### Bone turnover biomarkers

##### Rationale

Biochemical markers of bone turnover have been shown to be promising in predicting fractures in the elderly up to 2 years before the event [53]. Assessing serum BAP (a bone formation biomarker) and serum TRAP (a bone resorption biomarker) could provide a more thorough clinical evaluation of bone status than BMD in predicting skeletal response to an exercise program and in monitoring bone resorption changes following initiation of exercise as early as month 3 [54].

##### Methods

The concentration of bone formation biomarker, BAP, in serum was measured using Metra™ BAP immunoassay kits (Quidel Corporation, San Diego, CA). The intra-assay CV of the BAP assay was 5.2%, and the inter-assay CV was 5.0%. Such procedures are routinely performed in our laboratory [47]. The concentration of bone resorption biomarker, TRAP, in serum was quantified using a commercial kit with a monoclonal antibody specific for the serum band 5 tartrate-resistant acid phosphatase (MicroVue™ TRAP5b, Quidel). The intra-assay variability was 2.2% CV and inter-assay variability was 3.0% CV. In order to avoid the interassay variation, the samples from baseline, 4, 12, and 24 week visits of the same patients were measured for bone biomarkers withitn the same assay each time.

#### Urinary 8-OHdG level

##### Rationale

When ROS attack all macromolecules including lipids, proteins, and DNA, the addition of the hydroxyl group (-OH) to the C8-position of guanine produces C8-OH-adduct radical [55], which is subsequently converted to 8-OH-Guanine (8-OH-Gua) by a one-electron oxidation [56]. While damaged lipids and proteins can be removed by metabolic turnover of molecules, impaired DNA has to be repaired *in situ*, or destroyed by apoptotic processes, if not to result in mutations. In humans, 8-OH-Glu glycosylase is the primary enzyme for the repair of 8-OH-Gua in a short-patch base-excision repair [57]. The excised form of 8-OH-Gua is 8-OHdG is excreted into urine without further metabolism and is stable for a significant time. Thus, urinary 8-OHdG generally reflects the whole body's oxidative DNA damage and repair, and becomes a putative biomarker for oxidative stress [58]. Detection of urinary 8-OHdG provides a sensitive and non-invasive means to evaluate the efficacy of dietary antioxidant supplements, such as GTP. We measured urinary 8-OHdG levels in this study to evaluate the effect of treatments.

##### Methods

The concentration of 8-OHdG, DNA damage oxidative stress biomarker, in urine was determined [48]. The urinary 8-OHdG was extracted from 1 ml urine with the Oasis^® ^HLB 3 cc (60 mg) cartridge. The eluents were dried under ultra-pure N_2 _stream and reconstituted in buffer (10 mM ammonium acetate in 2% MeOH, pH 4.3) for analysis with the ESA HPLC-CoulArray system. The system consisted of double Solvent Delivery Modules (Model 582 pump), Autosampler (Model 542) with 4°C cool sample tray and column oven, CoulArray Electrochemical Detector (Model 5600A), and an Operating Computer. The HPLC column for 8-OHdG analysis was the Waters YMC basic™ column (S3 μm, 4.6 × 150 mm). The mobile phase consisted of buffer A (10 mM ammonium acetate, pH 4.3) and buffer B (methanol). Flow rate was kept at 0.8 ml/min and a linear gradient (0-40% MeOH in 15 min) was applied for chromatographic separation with the peak of 8-OHdG eluted at around 9.5 min. The CoulArray Detector was set at 270, 300, 330, and 360 mv; the highest peak appeared at 330 mv channel. Authentic standard 8-OHdG was used for qualification by retention times and response patterns, and quantification by calibration curves. The limit of detection for 8-OHdG was 1 ng/ml urine. The amount of 8-OHdG was adjusted by urinary creatinine level. Urinary creatinine level was determined colorimetrically with a Diagnostics Creatinine Kit (Sigma Co). Absorbance at 500 nm was recorded by a DU640 VIS/UV spectrophotometer [48].

#### Serum and Urinary GTP concentration

##### Rationale

The bioavailability of GTP was considered when we quantitatively evaluated the biological effects of GTP intervention. Therefore, we planed to measure the GTP levels in biofluid, such as serum and urine, of participants at baseline, 4, 12, and 24 weeks.

##### Methods

The procedures for analyzing serum and urinary GTP conjugates were modified from established protocols [48,59]. Briefly, serum (200 μL) or urine (500 μL) samples were incubated without enzyme, with betaglucuronidase (500 units for serum and 200 units for urine) or with 40-units of sulfatase at 37°C simultaneously for 1 h, to generate free GTP, free plus glucuronidated GTPs, free plus sulfated GTPs, and methylated GTPs, respectively. The extracted samples were vacuum-dried and reconstituted for HPLC-CoulArray analysis.

The HPLC-CoulArray system consists of double Solvent Delivery Modules (Model 582 pump), Autosampler (Model 542) with 4°C cool sample tray and column oven, CoulArray Electrochemical Detector (Model 5600A), and an Operating Computer. The HPLC column was an Agilent Zorbax reverse-phase column, Eclipse XDB-C_18 _(5 μm, 4.6 × 250 mm). The mobile phase included buffer A (30 mM NaH_2_PO_4_/ACN/THF = 98/1.8/0.2, pH 3.35) and buffer B (15 mM NaH_2_PO_4_/ACN/THF = 30/63/7, pH 3.45). Flow rate was set at 1 ml/min and the gradient started from 4% buffer B, to 24% B at 24 min, to 95% B at 35 min, kept at 95% until 42 min, dropped to 4% at 50 min, and maintained at 4% until 59 min. Authentic standards were prepared with ascorbic acid.

Calibration curves for individual GTP components were generated separately, and EGC, EC, EGCG and ECG were eluted at around 14, 21, 24 and 29 min, respectively. The electrochemical detector was set at -90, -10, 70 and 150 mV potentials, with the main peaks appearing at -10 mV (EGC), 70 mV (EC, EGCG) and 150 mV (ECG). Quality assurance and quality control procedures were taken during analyses, including analysis of authentic standards for every set of five samples and simultaneous analysis of spiked urine sample daily. The limits of detection were 0.5 ng/mL urine for EC, EGC, and methylated-EGC; and 0.5 ng/mL plasma for EGCG, ECG, and dimethylated-EGCG, respectively. The levels of glucuronidated or sulfated GTP or methylated GTP were calculated by subtraction of free GTP levels from corresponding digestions. The four forms of GTP were pooled as a total for calculation of conjugation percentiles.

### Statistical Analysis

Due to the longitudinal design of the proposed study, a model of repeated measurements with random effect error terms was used. Statistical software SAS was employed to conduct the analyses, controlling for the within subject correlation. First, the measurements were compared across the 4 groups at the baseline. In addition, participant characteristics were compared to detect whether participants in these four groups were different in certain characteristics. Second, the changes in the measurements from baseline to the follow-ups were calculated for each group. We then tested whether these changes were statistically significant. Time-dependent trends, if any, were identified. For between-group differences, a two-way ANCOVA (TC and GTP) was conducted and controlled for within-subject correlation. To control for the effect of age, body mass index, BMD, and other covariates (i.e., years after menopause, prior meditation and mind-body exercise exposure, habit of green tea consumption, etc), a multivariate ANCOVA was also performed. Third, the characteristics of participants who dropped out were compared with those of the participants who stayed for the entire study period, in order to detect potential biases.

## Discussion

We have presented the rationale, design, and methodology of a placebo-controlled randomized trial, with quantitative studies, to investigate a new complementary and alternative medicine strategy featuring a dietary supplement and a mind-body exercise for alleviating bone loss in postmenopausal women with low bone mass. The results of this research will be presented as soon as they are available.

## Competing interests

The authors declare that they have no competing interests.

## Authors' contributions

CLS obtained funding for the study. CLS, MCC, JKY, CKF, KTX, BCP, and JSW designed this trial. CLS wrote the first draft of the manuscript and the rest of coauthors participated in the revision of subsequent dra. All authors approved the final version of the manuscript. None of the authors declared any conflicts of financial interest.

## Pre-publication history

The pre-publication history for this paper can be accessed here:


